# Randomized controlled trial of ragweed sublingual immunotherapy tablet in the subpopulation of Canadian children and adolescents with allergic rhinoconjunctivitis

**DOI:** 10.1186/s13223-021-00626-2

**Published:** 2021-12-09

**Authors:** Anne K. Ellis, Remi Gagnon, David I. Bernstein, Hendrik Nolte

**Affiliations:** 1 Division of Allergy & Immunology, Department of Medicine, Queen’s University, Kingston, ON Canada; 2 Clinique Spécialisée en Allergie de La Capitale, Québec, QC Canada; 3 Division of Immunology, Allergy and Rheumatology, University of Cincinnati College of Medicine and Bernstein Clinical Research Center, Cincinnati, OH USA; 4 ALK, Bedminster, NJ USA

**Keywords:** Ragweed, Children, Adolescents, Allergic rhinoconjunctivitis, Sublingual immunotherapy

## Abstract

**Background:**

Post hoc analyses of randomized placebo-controlled trials have demonstrated efficacy and tolerability of the ragweed sublingual immunotherapy (SLIT)-tablet in Canadian adults with ragweed pollen-induced allergic rhinitis/conjunctivitis (AR/C). This post hoc analysis evaluated the efficacy and tolerability of the ragweed SLIT-tablet in the subpopulation of Canadian children and adolescents with AR/C in a previously described randomized, double-blind, placebo-controlled trial.

**Methods:**

The trial (NCT02478398) was conducted in North American and European children/adolescents ages 5–17 years with ragweed pollen-induced AR/C with or without asthma (FEV_1_ ≥ 80% predicted). Participants were randomized to daily ragweed SLIT-tablet (12 Amb a 1-U) or placebo for up to 28 weeks. The primary endpoint was the average total combined score (TCS; sum of rhinoconjunctivitis daily symptom score [DSS] and daily medication score [DMS]) during peak ragweed pollen season (RPS). Key secondary endpoints were TCS during the entire RPS, and DSS and DMS during peak RPS. Post hoc analyses of the primary and key secondary endpoints were conducted in the subpopulation of Canadian participants.

**Results:**

Of the 1025 randomized participants, 246 (SLIT-tablet, n = 116; placebo, n = 130) were in the Canadian subpopulation. In the total study population, relative TCS (95% CI) improvement with ragweed SLIT-tablet versus placebo was − 38.3% (− 46.0%, − 29.7%; least square [LS] mean difference, − 2.73; P < 0.001) during peak RPS. In the Canadian subpopulation, relative TCS improvements with ragweed SLIT-tablet versus placebo were − 40.8% (− 54.5%, − 20.2%; LS mean difference, − 1.59; P = 0.001) during peak RPS and − 36.6% (− 50.2%, − 16.5%; LS mean difference, − 1.36; P = 0.002) during the entire RPS. DSS and DMS during peak RPS in the Canadian subpopulation improved with SLIT-tablet versus placebo by − 30.6% (− 45.2%, − 7.7%; LS mean difference, − 0.94; P = 0.010) and − 77.2% (− 97.5%, − 44.2%; LS mean difference, − 0.66; P = 0.003), respectively. No events of anaphylaxis, airway compromise, intramuscular epinephrine administration, eosinophilic esophagitis, or severe treatment-related systemic allergic reactions were reported in the overall population or Canadian subpopulation.

**Conclusion:**

Efficacy and safety of the ragweed SLIT-tablet in Canadian children/adolescents with ragweed pollen-induced AR/C was consistent with the total study population. The ragweed SLIT-tablet resulted in clinically meaningful improvement in symptoms, decreased symptom-relieving medication use, and was well tolerated in Canadian children/adolescents.

*Trial registration*: clinicaltrials.gov, NCT02478398. Registered June 23, 2015, https://clinicaltrials.gov/ct2/show/NCT02478398?term=NCT02478398&draw=2&rank=1

**Supplementary Information:**

The online version contains supplementary material available at 10.1186/s13223-021-00626-2.

## Introduction

Allergic rhinitis/conjunctivitis (AR/C) can cause a substantial burden on children and adolescents, interfering with sleep and daily activities, as well as negatively impacting school attendance and performance [1, 2]. Ragweed pollen is a common reported cause of AR/C and sensitization to ragweed has increased as the plant distribution has spread [2–4]. Although published data on the sensitization to ragweed in Canada is generally lacking, the average prevalence of ragweed sensitization in adults in the general population across Canada was 15%, but was as high as 33% in Montreal [5]. A small (N = 39), single-site study of 6-year old Canadian children indicated that 15% were sensitized to ragweed (unpublished observations, Anne K. Ellis). In the US, 11% of children ages 6–9 years and 19% of children and adolescents ages 10–19 years are sensitized to ragweed [6].

Allergy immunotherapy (AIT) delivered either by subcutaneous injection (SCIT) or sublingual tablets is a recommended treatment for AR/C [7]. Unlike symptom-relieving medications, AIT is disease-modifying with persisting effects and the potential to prevent the development of allergic asthma in children [8, 9]. The sublingual immunotherapy (SLIT)-tablet form of AIT administration is an appealing alternative to SCIT for children, adolescents, and their caregivers because it can be self-administered at home after the first dose, it avoids the need for repeat injections, and it has a better safety profile than SCIT [10–12].

Post hoc analyses of randomized, double-blind, placebo-controlled trials [13, 14] have demonstrated the efficacy and tolerability of the 12 Amb a 1-U ragweed SLIT-tablet (Ragwitek/Ragwizax, ALK, Hørsholm, Denmark) in Canadian adults with ragweed pollen-induced AR/C [15]. The objective of this post hoc analyses of a previously described randomized, double-blind, placebo-controlled trial [16] of the ragweed SLIT-tablet conducted in children and adolescents was to determine the efficacy and tolerability of the ragweed SLIT-tablet in the subpopulation of Canadian children and adolescents.

## Methods

Details of the original randomized, double-blind, placebo-controlled, phase 3 trial (P008; clinicaltrials.gov identifier NCT02478398; EudraCT: 2014-004341-27) conducted in North America and Europe have been previously reported [16]. Briefly, participants aged 4 to 17 years with a history of physician-diagnosed ragweed pollen-induced AR/C with or without asthma (forced expiratory volume in 1 s ≥ 80%) were randomized 1:1 to once-daily ragweed SLIT-tablet (12 Amb a 1-U dose; ALK, Hørsholm, Denmark) or placebo. To complete the enrollment goal, 3 separate cohorts were recruited over 3 consecutive (2016–2018) ragweed pollen seasons (RPS). Treatment was started approximately 12 to 20 weeks before the RPS and continued for the duration of the season (approximately 8 weeks). The first dose was administered at the study site and subsequent doses were administered at home. Open-label symptom-relieving medication was provided to all participants (see Additional file 1: Table S1) and a short-acting beta-agonist was provided to participants with asthma. Self-injectable epinephrine was provided at 2 sites in Canada per Institutional Review Board request.

The study was approved by each sites’ Institutional Review Board and was conducted in compliance with the Declaration of Helsinki and Good Clinical Practice. The guardian of each participant provided written informed consent before the participant started the trial.

### Ragweed pollen season

The RPS for each study site was defined as starting on the first day of 3 consecutive recorded days with a *Ambrosia artemisiifolia* pollen count of ≥ 10 grains/m^3^ and ending on the last day of the last 3 consecutive recorded days with a pollen count of ≥ 10 grains/m^3^. Peak RPS was the 15 consecutive recorded days within the RPS with the highest 15-day moving average pollen count.

### Study assessments

Participants (or their guardians) self-recorded allergy symptoms and symptom-relieving medication use in a daily e-diary. The average total combined symptom and medication score (TCS) during the peak RPS was the primary endpoint. The TCS is the sum of the average rhinoconjunctivitis daily symptom score (DSS) and average rhinoconjunctivitis daily medication score (DMS). The key secondary endpoints were the TCS during the entire RPS, DSS during the peak RPS, and DMS during the peak RPS. DSS and DMS during the entire RPS were also evaluated. The DSS was calculated from participant (or guardian) daily scoring of 6 rhinoconjunctivitis symptoms (runny nose, stuffy nose, sneezing, itchy nose, itchy eyes, and watery eyes) on a scale of 0 (no symptoms) to 3 (severe symptoms) and DMS was calculated based on participant (or guardian) recording of symptom-relieving medication use (Additional file 1: Table S1).

Safety was assessed by monitoring of adverse events (AEs) and by participant (or guardian) recording of solicited and pre-selected local AEs [17] that occurred within the first 60 min after treatment on a SLIT Report Card [18] for approximately the first 28 days of treatment. Percent compliance was calculated for each participant as the number of doses taken divided by the number of days between first tablet intake and date of last tablet intake. On average, treatment compliance in the trial was at least 90% for 73% of participants, and 98% were at least 75–90% compliant.

### Statistical analysis

Post hoc analyses of the primary and key secondary efficacy endpoints were conducted in the subpopulation of Canadian participants in the Full Analysis Set (FAS), using the same statistical methods used for the full study population that were pre-specified in the protocol. The FAS was defined as all participants who received at least one dose of study treatment and with at least one diary record during the peak RPS. Post hoc safety analysis was conducted in the subpopulation of Canadian participants in the All-Subjects-as-Treated dataset, defined as all randomized participants who received at least one dose of study treatment. Least square (LS) mean scores, differences in LS mean scores, and 2-sided 95% CI for the efficacy endpoints were calculated using an analysis of variance (ANOVA) model which included fixed effects of treatment, baseline asthma status (yes/no), age group (5–11 years or 12–17 years), pollen season, and pollen region nested within pollen season. The percentage treatment differences in LS means with ragweed SLIT-tablet relative to placebo were calculated. Corresponding confidence intervals were calculated by the bootstrap method using 10,000 iterations. No missing data were imputed. Statistical analyses were conducted using SAS version 9.4 (Cary, NC).

## Results

### Participants

**Fig. 1:**
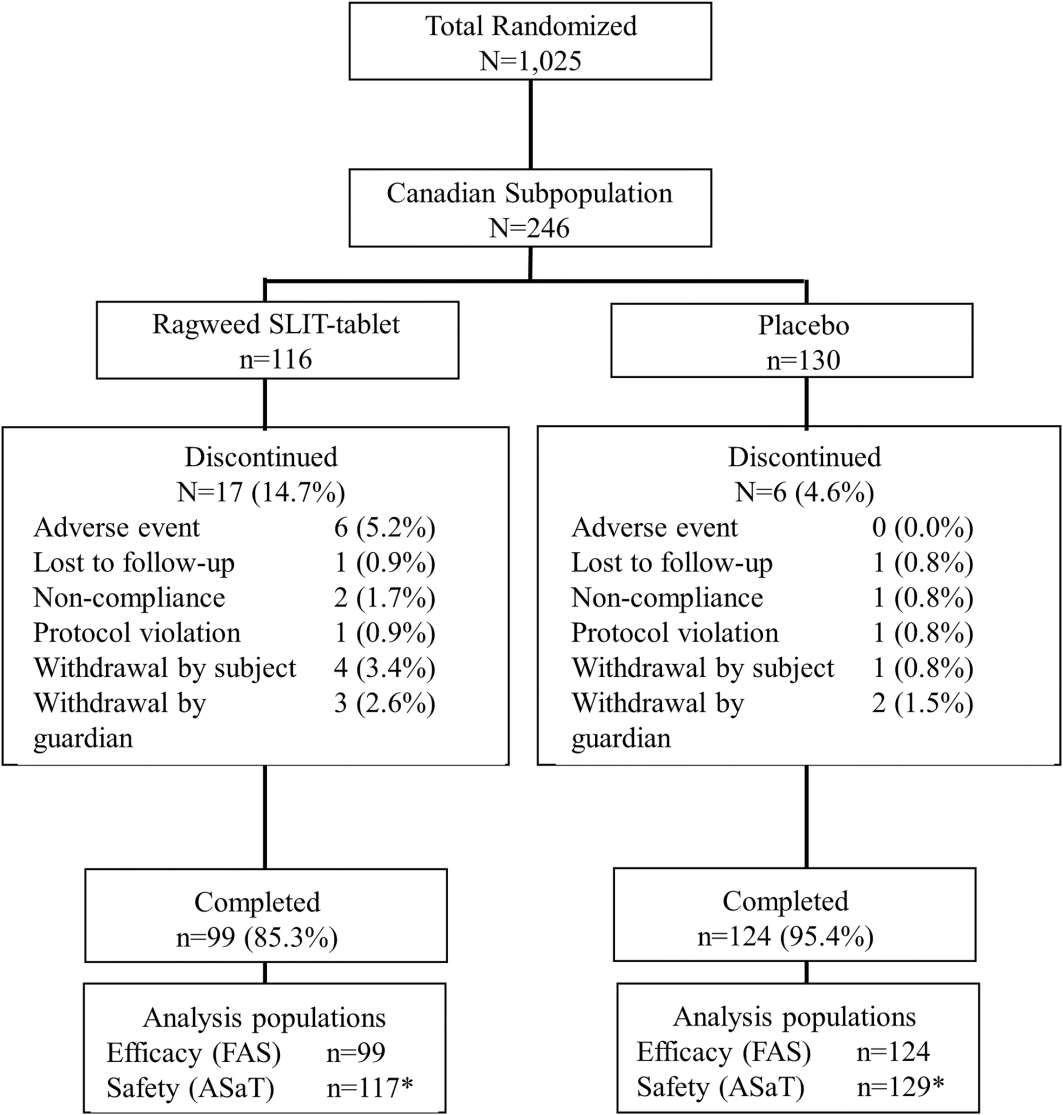
Subject disposition in the Canadian subpopulation. ASaT, all subjects as treated; FAS, full analysis set; SLIT, sublingual immunotherapy. *One subject randomized to placebo received the ragweed SLIT-tablet by mistake

Of the total 1025 participants randomized, 246 of the participants were Canadian (24% of the entire study population) and were analyzed in the safety population, and 223 completed the trial and were included in the FAS (Fig. 1). Discontinuation rates in the Canadian subpopulation were 14.7% with the ragweed SLIT-tablet and 4.6% with placebo (Fig. 1).

Baseline demographics and clinical characteristics in the Canadian subpopulation were well balanced between the treatment groups (Table 1) and were similar to the total study population [16]. The majority of Canadian participants were male (58.9%) and white (87.8%); the mean age was 12.4 years and 60.6% were aged 12 to 17 years. Asthma was reported in 35.8% of participants and most (90.7%) were polysensitized. The majority (82.5%) of Canadian participants had allergic rhinoconjunctivitis, whereas 17.5% had only allergic rhinitis.

**Table 1:** Baseline characteristics and demographics of randomized participants in the Canadian subpopulation

	Ragweed SLIT-Tablet (n = 116)	Placebo (n = 130)
Male, n (%)	67 (57.8)	78 (60.0)
Age, mean (SD) y	12.5 (3.2)	12.4 (3.2)
< 12 y, n (%)	46 (39.7)	51 (39.2)
≥ 12 y, n (%)	70 (60.3)	79 (60.8)
White, n (%)	101 (87.1)	115 (88.5)
Participants with asthma, n (%)	38 (32.8)	50 (38.5)
IgE sensitization type, n (%)		
Ragweed only	9 (7.8)	14 (10.8)
Ragweed and other allergens	107 (92.2)	116 (89.2)
Alder tree	68 (58.6)	74 (56.9)
*Alternaria tenuis*	47 (40.5)	47 (36.2)
Birch tree	74 (63.8)	81 (62.3)
Cat dander	74 (63.8)	94 (72.3)
*Cladosporium herbarum*	28 (24.1)	26 (20.0)
Dog dander	64 (55.2)	80 (61.5)
House dust mites	48 (41.4)	60 (46.2)
Mugwort	65 (56.0)	78 (60.0)
Timothy grass	70 (60.3)	85 (65.4)
*A artemisiifolia* SPT wheal size, mean (SD) mm	10.2 (3.0)	10.5 (3.5)
*A artemisiifolia* serum specific IgE, mean (SD) kU_A_/L	17.7 (21.5)	20.5 (26.1)

*SLIT* sublingual immunotherapy, *SPT* skin prick test

### Ragweed pollen seasons

In Canada, the mean (SD) *Ambrosia artemisiifolia* pollen counts in 2016, 2017, and 2018 during the peak RPS were 70 (36) grains/m^3^, 66 (24) grains/m^3^, and 109 (47) grains/m^3^, respectively, and during the entire RPS were 43 (19) grains/m^3^, 44 (14) grains/m^3^, and 68 (28) grains/m^3^.

### Average daily TCS

**Fig. 2:**
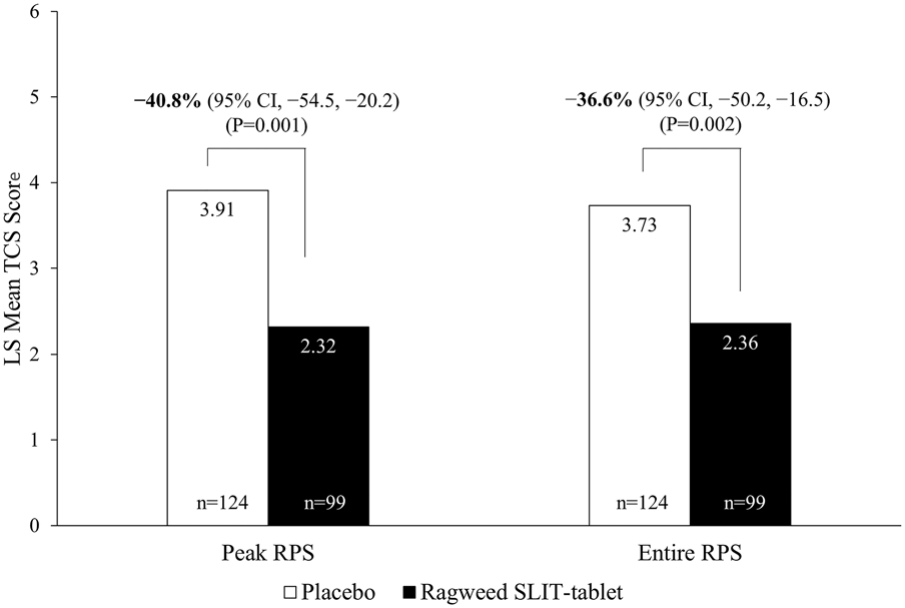
Total combined score (TCS) in the Canadian subpopulation during the peak and entire ragweed pollen season (RPS) (FAS population). FAS, full analysis set; SLIT, sublingual immunotherapy

In the total study population, relative TCS (95% CI) improvement with ragweed SLIT-tablet versus placebo was − 38.3% (− 46.0%, − 29.7%; LS mean difference, − 2.73; P < 0.001) during peak RPS. In the Canadian subpopulation, relative TCS (95% CI) improvement with ragweed SLIT-tablet compared with placebo was − 40.8% (− 54.5%, − 20.2%; Fig. 2), with a LS mean score difference of − 1.59 (P = 0.001; Table 2) during peak RPS. Average TCS during the entire RPS improved with ragweed SLIT-tablet compared with placebo by − 36.6% (− 50.2%, − 16.5%; Fig. 2), with a LS mean score difference of − 1.36 (P = 0.002; Table 2).

**Table 2:** Total combined score (TCS), daily symptom score (DSS), and daily medication score (DMS) in the Canadian subpopulation during the peak and entire ragweed pollen season (FAS population)

	Peak ragweed pollen season		Entire ragweed pollen season	
Scale	Ragweed SLIT-tablet	Placebo	Ragweed SLIT-tablet	Placebo
TCS, LS mean	2.32	3.91	2.36	3.73
n	99	124	99	124
Difference vs placebo (95% CI)	− 1.59 (− 2.54, − 0.65)	–	− 1.36 (− 2.21, − 0.51)	–
Reduction vs placebo, % (95% CI)	− 40.8 (− 54.5, − 20.2)	–	− 36.6 (− 50.2, − 16.5)	–
P value	0.001	–	0.002	–
DSS, LS mean	2.12	3.06	2.12	2.89
n	99	125	99	125
Difference vs placebo (95% CI)	− 0.94 (− 1.65, − 0.22)	–	− 0.77 (− 1.43, − 0.11)	–
Reduction vs placebo, % (95% CI)	− 30.6 (− 45.2, − 7.7)	–	− 26.6 (− 41.6, − 3.9)	–
P value	0.010	–	0.022	–
DMS, LS mean	0.20	0.86	0.24	0.84
n	99	124	99	124
Difference vs placebo (95% CI)	− 0.66 (− 1.10, − 0.23)	–	− 0.59 (− 0.95, − 0.24)	–
Reduction vs placebo, % (95% CI)	− 77.2 (− 97.5, − 44.2)	–	− 70.8 (− 88.1, − 43.5)	–
P value	0.003	–	0.001	–

*FAS* full analysis set, *LS* least square, *SLIT* sublingual immunotherapy

### Average daily DSS and DMS

**Fig. 3:**
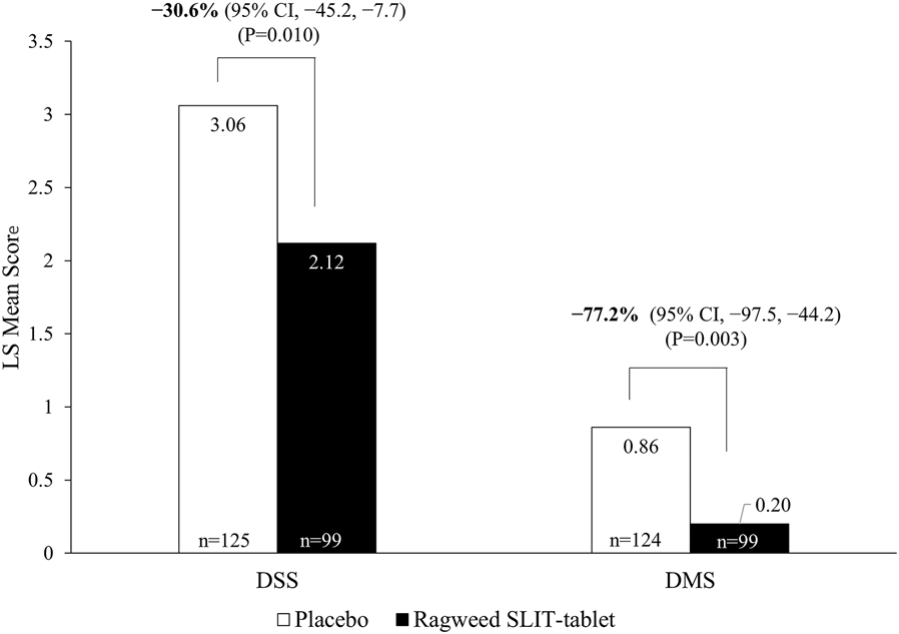
Daily symptom score (DSS) and daily medication score (DMS) in the Canadian subpopulation during the peak ragweed pollen season (FAS population). FAS, full analysis set; SLIT, sublingual immunotherapy

During peak RPS in the Canadian subpopulation, DSS and DMS (95% CI) were improved with ragweed SLIT-tablet compared with placebo by − 30.6% (− 45.2%, − 7.7%; LS mean difference, − 0.94; P = 0.010) and − 77.2% (− 97.5%, − 44.2%; LS mean difference, − 0.66; P = 0.003), respectively (Fig. 3 and Table 2). In all, 85.9% of Canadian participants receiving ragweed SLIT-tablet and 71.8% of participants receiving placebo had no symptom-relieving medication use (DMS = 0) during the peak RPS. Significant relative improvements in DSS and DMS were also observed during the entire RPS (Table 2).

### Safety

AEs were monitored until 14 days after the end of the trial. The ragweed SLIT-tablet was well tolerated in the overall population and Canadian subpopulation, with no deaths, anaphylaxis events, or intramuscular epinephrine administrations related to ragweed SLIT-tablet treatment. There were also no severe events of ragweed SLIT-tablet-related local swellings of the mouth or throat, systemic allergic reactions, or asthma events. There were no reported events of eosinophilic esophagitis. The rate of discontinuation with ragweed SLIT-tablet due to AEs in the Canadian subpopulation was 5.1% with the ragweed SLIT-tablet and 0.0% with placebo (Table 3).

**Table 3:** Safety summary in the Canadian subpopulation (All-Subjects-as-Treated)

AE, No. (%)	Ragweed SLIT-tablet (n = 117)*	Placebo (n = 129)*
Treatment-emergent AE	108 (92.3)	96 (74.4)
Treatment-related AE	89 (76.1)	47 (36.4)
SAE	0	0
Treatment-related SAE	0	0
AE leading to treatment discontinuation	6 (5.1)	0
Treatment-related AE leading to treatment discontinuation	6 (5.1)	0
Discontinued treatment due to SAE	0	0
Discontinued treatment due to a treatment-related SAE	0	0

*AE* adverse event, *SAE* serious adverse event

^*^One subject randomized to placebo received the ragweed SLIT-tablet by mistake

In addition to AE monitoring, the occurrence of specific local AEs was actively solicited in the SLIT Report Card. Treatment-related AEs in the Canadian subpopulation were reported by 76.1% of participants receiving ragweed SLIT-tablet and 36.4% of participants receiving placebo (Table 3). No serious treatment-related AEs were reported in the Canadian subpopulation (Table 3), although 2 serious AEs related to ragweed SLIT-tablet occurred in the overall study population and have been previously described [16]. One Canadian participant experienced a mild, non-serious systemic allergic reaction (skin pruritus, redness, eye swelling/pruritus, sneezing, runny nose) beginning on day 6 and discontinued the study on day 34. Additional details of this systemic allergic reaction have been previously described [16].

## Discussion

The results of this post hoc analyses indicate that the ragweed SLIT-tablet improved symptoms and reduced symptom-relieving medication use in Canadian children and adolescents with AR/C during the peak and entire RPS. The efficacy and tolerability of the ragweed SLIT-tablet in the subpopulation of Canadian participants was consistent with the full study population that included participants from North America and Europe. Clinically meaningful benefit of the ragweed SLIT-tablet in the Canadian subpopulation was demonstrated, with a 40.8% improvement in the TCS over placebo during the peak RPS, despite the allowed use of symptom-relieving rescue medications in both the active and placebo groups. The impressive reduction in the need for symptom-relieving rescue medication (DMS, − 77%) during the peak season suggests that individuals with severe symptoms may show substantial clinical benefit from SLIT-tablet treatment. The magnitude of TCS improvement in the Canadian children and adolescents was similar to that of Canadian adults (39.5%; p < 0.0001 vs placebo) in a post hoc analysis of 2 adult ragweed SLIT-tablet trials. [15].

The overall safety profile was similar between the Canadian subpopulation and the total study population. The proportion of Canadian participants with treatment-emergent or treatment-related AEs (92.3% and 76.1%, respectively) in the ragweed SLIT-tablet group was also comparable to that in the Canadian adult subpopulation (94.5% and 80.9%, respectively) [15]. The rates of AEs were slightly higher during the trial than for most previous trials of SLIT-tablets in both the placebo and ragweed-SLIT-tablet groups, which is most likely because of the use of the SLIT Report Card. The SLIT Report Card is a questionnaire that actively solicits the patient-reported occurrence of specific local AEs that are commonly associated with SLIT [18] and its use has been shown to increase the frequency of many of these AEs compared with standard, open-ended AE reporting used in previous SLIT-tablet trials [19]. Practically, AEs did not appear to be particularly problematic for participants as the proportion of Canadian children and adolescents receiving the ragweed SLIT-tablet who discontinued due to treatment-related AEs was 5.1%. This rate is lower than that observed in Canadian adults receiving the ragweed SLIT-tablet (12.7%) [15] and is similar to rates of discontinuation observed with grass and house dust mite SLIT-tablet [20]. The majority of the remaining discontinuations in the ragweed SLIT-tablet group in the Canadian participants were withdrawals by the subject or guardian. Importantly, approximately a third of the Canadian subpopulation had mild to moderate stable asthma, yet there were no severe asthma events.

A strength of this analysis is the large size of the analyzed subpopulation. The trial was the largest pediatric SLIT-tablet trial conducted to date and therefore provided opportunity for robust subpopulation analyses. Even within this smaller subpopulation, the improvement in TCS met the various definitions of a clinically meaningful effect, including those of regulatory agencies and professional organizations [21, 22]. The results and conclusions from this post hoc analysis are limited to those in the Canadian subpopulation and cannot be generalized to other regional subpopulations.

The ability to deliver the ragweed SLIT-tablet at home may be ideal for children, adolescents, and caregivers for whom it may be difficult to schedule office visits for SCIT administration around school and work schedules and also for those who live in remote areas. In addition, patients may have negative feelings about injections, which are avoided with the use of the SLIT-tablet [10] Thus, the efficacy and tolerability demonstrated in the current analysis support the use of the ragweed SLIT-tablet in Canadian children and adolescents with AR/C as an alternative to SCIT.

## Supplementary Information

**Additional file 1:** **Table S1.** Scoring of symptoms and medication use.

## Data Availability

All data generated or analyzed during this study are included within this published article (and its Additional file 1).

## Abbreviations

AE: Adverse event
AR/C: Allergic rhinitis with or without conjunctivitis
DMS: Daily medication score
DSS: Daily symptom score
RPS: Ragweed pollen season
SCIT: Subcutaneous immunotherapy
SLIT: Sublingual immunotherapy
TCS: Total combined score
