# Supplementary material for: Randomized controlled trial of ragweed sublingual immunotherapy tablet in the subpopulation of Canadian children and adolescents with allergic rhinoconjunctivitis
Source: Allergy Asthma Clin Immunol. 2021 Dec 9;17:127. doi: 10.1186/s13223-021-00626-2 (PMC8656080; doi:10.1186/s13223-021-00626-2)
Supplement: Supplementary file 1 — Additional file 1: Table S1. Scoring of symptoms and medication use. [file 13223_2021_626_MOESM1_ESM.docx]

**Supplemental Material**

**Table S1.** Scoring of symptoms and medication use.

| **Symptom** | **Score** | | **Maximum Daily Score** |
| --- | --- | --- | --- |
| Runny nose | 0=none  1=mild  2=moderate  3=severe | | 3 |
| Stuffy nose |  |  | 3 |
| Sneezing |  |  | 3 |
| Itchy nose |  |  | 3 |
| Itchy eyes |  |  | 3 |
| Watery eyes |  |  | 3 |
| Maximum rhinoconjunctivitis DSS | | | 18 |
| **Symptom-relieving Medication** | **Subject Dosing Instructions** | **Score/Dose Unit** | **Maximum Daily Score** |
| Loratadine, 1 mg/mL syrup or 10 mg tablet | 5 years old: 5 mL daily  6–17 years old: 10 mL or 1 tablet daily | 6 | 6 |
| Olopatadine hydrochloride, 0.1% | 1 drop per eye, twice daily | 1.5 per drop | 6 |
| Mometasone nasal spray, 50 μg/spray | 5-11 years old: 1 spray in each nostril daily | 4 per spray | 8 |
|  | 12-17 years old: 2 sprays in each nostril daily | 2 per spray |  |
| Maximum rhinoconjunctivitis DMS | | | 20 |
| **Maximum TCS^*^** |  |  | **38** |

DMS, daily medication score; DSS, daily symptom score; TCS, total combined score.

^*^Sum of rhinoconjunctivitis DSS and rhinoconjunctivitis DMS.
